# Effects of 2,3,7,8-Tetrachlorodibenzo-p-dioxin on T Cell Differentiation in Primary Biliary Cholangitis

**DOI:** 10.1155/2020/1754975

**Published:** 2020-08-24

**Authors:** Chunhui She, Jing Wang, Ning Tang, Zhaoyang Liu, Lishan Xu, Bin Liu

**Affiliations:** Department of Rheumatology, Affiliated Hospital of Qingdao University Qingdao, Shandong Province 266001, China

## Abstract

Exposure to dioxins, such as 2,3,7,8-tetrachlorodibenzo-p-dioxin (TCDD), is reported to affect the autoimmune system and increase the risk of autoimmune disease. Generally, dioxin exerts its toxicity via aryl hydrocarbon receptor (AhR). Primary biliary cholangitis (PBC) is a chronic autoimmune disease, and its pathogenesis involves the interplay between immune and environmental factors. This study showed the effect of dendritic cells (DCs) activated by TCDD on naïve CD4^+^ T cell differentiation in patients with PBC. CD14^+^ mononuclear cells were isolated from peripheral blood mononuclear cells (PBMCs) of patients with PBC and healthy people by magnetic cell separation and introduced into DCs. Two days after stimulation by TCDD, DCs were cocultured with naïve CD4^+^ T cells in a ratio of 1 : 2 for 3 days. Then, differentiation-related factors for naïve CD4^+^ T cells were detected by real-time fluorescence quantitative polymerase chain reaction, enzyme-linked immunosorbent assay, and flow cytometry. The results showed that TCDD-activated DCs could promote Th1 and Th17 differentiation in patients with PBC. Therefore, this study demonstrated TCDD as an AhR agonist in regulating naïve CD4^+^ T cell differentiation in patients with PBC.

## 1. Introduction

Primary biliary cholangitis (PBC) is a chronic autoimmune disease most commonly observed in female patients. It is clinically characterized by antimitochondrial antibody (AMA) positivity, increased levels of alkaline phosphatase (ALP), and damage to both small- and medium-sized bile ducts due to immune infiltration [1]. The pathogenesis of PBC involves the interplay between immune and environmental factors [2].

Dioxin-like compounds (DLCs) are colorless, odorless, and highly toxic environmental pollutants, derived mainly from the incineration of municipal and industrial wastes; of these compounds, 2,3,7,8-tetrachlorodibenzo-p-dioxin (TCDD) is the most toxic [3, 4]. They are also a new type of environmental organic pollutants, which have attracted increasing attention from all walks of life in recent years. Previous studies showed that DLCs exert their effect through the activation of the aryl hydrocarbon receptor (AhR) pathway [5]. AhR is a ligand-dependent transcription factor involved in body's response to the external biological environment via an important interaction between immunity and the environment [6]. TCDD is a class of synthetic, organic chlorine compound derived from biphenyl. A majority of toxicological effects elicited by coplanar TCDD exposure are associated with the activation of AhR and subsequent induction of responsive genes [7]. AhR agonist TCDD reduced antioxidant protection in exposed populations and led to adverse effects, such as immune disorders [8]. In addition, TCDD is a common environmental pollutant that induces autoimmunity independently of AhR [9].

DCs link innate and adaptive immune responses to various environmental pollutants and are critical in antigens presenting to CD4^+^ T cells [10]. Active forms of cytokines and chemokines trigger a series of inflammatory cascades leading to the differentiation of CD4^+^ T cells, including autoimmunity [11]. Patients with PBC have impaired T cell functions, which may cause damage to both small- and medium-sized bile ducts due to immune infiltration [12]. Therefore, understanding the balance between the beneficial and pathological roles of these cytokines during inflammation is a key to understanding PBC pathogenesis.

This study was performed to explore the effects of AhR on naïve CD4^+^ T cell differentiation in patients with PBC and the role of immune cell abnormalities caused by environmental factors in the pathogenesis of PBC.

## 2. Materials and Methods

### 2.1. Materials

The following materials were used in the study: lymphocyte separation fluid (Ficoll-Paque Premium, 1.077 ± 0.001 g/ml, GE Healthcare, USA), magnetic column separation (MS columns, Miltenyi Biotec, Bergisch Gladbach, Germany), CD14 MicroBeads (Miltenyi Biotec), granulocyte-macrophage colony-stimulating factor (GM-CSF) (Peprotech, USA), interleukin- (IL-) 4 (Peprotech), TCDD (Sigma, USA), CH223191 (Sigma), RNAiso Plus (TaKaRa, Dalian, China), OneStep PrimeScript Real-Time Polymerase Chain Reaction (RT-PCR) Kit (TaKaRa), SYBR Green master mix (TaKaRa), CCK-8 (Solarbio, China, Enzyme-Linked Immunosorbent Assay (ELISA) kit (Cloud-Clone Corp., Wuhan, China), and antibodies (Cell Signaling Technology). All fluorescein-conjugated and isotype-matched antibodies were purchased from BD Biosciences (CA, USA). Antibodies were obtained from Cell Signaling Technology.

### 2.2. CD14^+^ Mononuclear Cells Introduced into DCs

This study was approved by the ethics committee of the Affiliated Hospital of Qingdao University, and written informed consent forms were obtained (approval number: QYFY WZLL 25571). Peripheral blood mononuclear cells (PBMCs) were collected from 10 patients with PBC (PBC group) and 10 healthy persons (healthy control group, HC) matched for age and sex using lymphocyte separation fluid. Patients with PBC were all new, meeting two of the following three criteria: (1) biochemical evidence of intrahepatic cholestasis with ALP ≥ normal upper limit for ≥6 months, (2) serum titer of AMA ≥ 1 : 40, and (3) liver histology compatible with PBC characteristics, characterized by nonsuppurative cholangitis and granulomatous destruction of the interlobular bile ducts (drug-induced liver injury needed to be excluded) [13]. At the same time, the PBC patients should exclude liver damage, infectious diseases, and other autoimmune diseases, as well as the absence of other complications, such as Sjogren's syndrome, thyroid disease, and rheumatoid arthritis, and the PBC patients did not use drugs such as UDCA or immunosuppressive therapy before collecting peripheral blood. Subsequently, biochemical data for PBC patients and healthy controls were recorded (Table S1). The CD14^+^ monocytes were isolated from PBMCs using CD14 MicroBeads following the manufacturer's protocols. CD14^+^ monocytes were isolated from PBMCs of patients with PBC and HCs and stimulated with human GM-CSF (20 ng/ml) and IL-4 (20 ng/ml) for 6 days [14].

### 2.3. TCDD-Treated DCs and CD4^+^ T Cells Coculture

CH223191 is an antagonist of dioxin-induced AhR activation [15]. DCs were pretreated with 10 *μ*M CH223191 for 3 h, and TCDD was added as a negative group. DCs were divided into three groups: untreated DCs as a blank control, 10 nM TCCD-treated DCs as an experimental group (TCDD group), and 10 *μ*M CH223191+10 nM TCDD-treated DCs as a negative control group (CH+TCDD group). Naïve CD4^+^ T cells were also isolated from PBMCs of patients with PBC and HCs using naïve CD4^+^ T cell MicroBeads following the manufacturer's protocols. DCs were cocultured with naïve CD4^+^ T cells from patients with PBC or HCs in 96-well plates for 3 days. DCs from four groups, including patients with PBC or HCs, were cocultured with CD4^+^ T cells from patients with PBC or HCs at a rate of 1 : 2. As CD4^+^ T cells are nonadherent but DCs adhere to culture dishes, CD4^+^ T cells were collected from the supernatants and total RNA was isolated.

### 2.4. Flow Cytometry

CD14^+^ monocytes were collected and labeled for 30 min at 4°C in the dark with the following monoclonal antibodies (mAbs): anti-human CD14-fluorescein isothiocyanate (FITC), anti-human CD86-peridinin chlorophyll-cyanin 5·5 (PerCP-Cy5·5), anti-human CD80- phycoerythrin (PE), and anti-human CD40- phycoerythrin (PE). The cells were then washed, resuspended, and subjected to FACS analysis. The DC surface markers were researched using the following mAbs: anti-human MHC-II-PerCP-Cy5·5 and anti-human CD86-PE. The naïve CD4^+^ T cell surface markers were researched using the following mAbs: anti-human CD4-APC and CD45RA-FITC. After coculture, naïve CD4^+^ T cell differentiation was detected by labeling with anti-IL-4-PerCP-Cy5·5, anti-interferon- (IFN-) *γ*-PE, anti-IL-4-APC, anti-IL-17-PE, and anti-Foxp3-APC.

The FACS Canto II instrument (BD Immunocytometry Systems, CA, USA) was used for data acquisition. Data were analyzed with Diva-8 (BD Immunocytometry Systems) and FlowJo (Tree Star, OR, USA) software. Data were expressed as the percentage difference compared with isotype control using the mean fluorescence intensity (MFI) and cell ratio of each marker. FACS settings were identical for each sample analyzed.

### 2.5. RNA Isolation and Real-Time Fluorescence Quantitative Polymerase Chain Reaction

Total RNA was isolated from cells using RNAiso Plus. The cDNA was synthesized from 2.5 *μ*g RNA using a commercial OneStep PrimeScript RT-PCR Kit. Real-time PCR was monitored online using Roche 480 (Roche, USA) and SYBR Green master mix. The primer sequences are shown in Table 1. The relative gene expression was normalized to GAPDH and calculated using the 2^-*ΔΔ*CT^ method, where CT is the cycle threshold.

### 2.6. Detection of the Proliferation Ability of T Cells Using CCK-8

The cocultured naïve CD4^+^ T cells were cultured at 37°C for 3 days, and 10 *μ*l/well CCK-8 detection reagents were added to the 96-well culture plate. After incubation for 2 h at 37°C, the OD value at 450 nm was measured.

### 2.7. Enzyme-Linked Immunosorbent Assay

The protein levels were verified using an ELISA kit to explore further the change in T-bet, GATA-3, and ROR*γ*t after DCs were cocultured with CD4^+^ T cells. DCs were cocultured with naïve CD4^+^ T cells from patients with PBC or HCs in 96-well plates for 3 days. The supernatants were harvested, and the levels of cytokines (T-bet, GATA-3, and ROR*γ*t) in the supernatants were measured using the ELISA kit following the manufacturer's protocols.

### 2.8. Western Blot Analysis

Total proteins were extracted from DCs in both groups (the HC and PBC groups). DCs of each group were divided into the control, TCDD, and CH223191+TCDD groups. The total proteins were tested three times using the Western Blot analysis to detect the protein expression of AhR (1 : 1000) and CYP1A1 (1 : 1000). Proteins were quantified with ImageJ software and GraphPad Prism 7. GAPDH served as a loading control.

### 2.9. Statistical Analysis

Data were analyzed using GraphPad Prism version 7.0 software. All experiments were repeated on a minimum of three occasions. Data were expressed as mean ± standard error of mean. Group comparisons were performed using an unpaired, two-tailed Student *t* test. Multiple group comparisons were performed through analysis of variance (ANOVA). A two-way ANOVA was used when data with more than one factor were analyzed. *p* values less than 0.05 were considered statistically significant.

## 3. Results

### 3.1. CD14^+^ Mononuclear Cells Introduced into DCs

**Figure 1:**
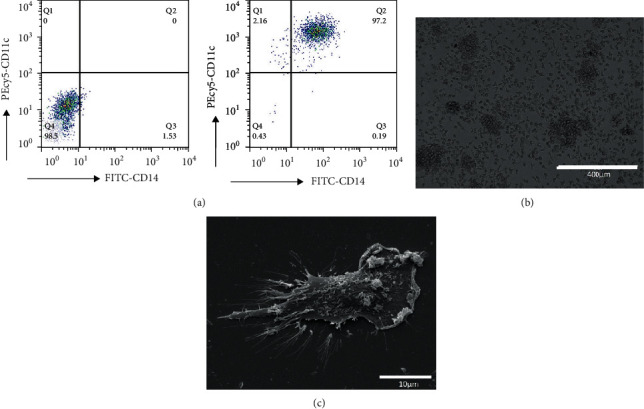
CD14^+^ monocytes were inducted to DCs. (a) The DC surface markers CD11c and CD14 were detected by FACS and the proportion of CD11c^+^ CD14^+^ DCs reached 97.2% after GM-CSF and IL-4 treatment. (b) Under microscope, the cells were irregular in shape, including stars, circles, and spindles, and the burrs process further increased and were in typical DCs morphology. (c) Under scanning electron microscope, the cell bodies showed an irregular shape and rough surface, and the microvilli on the cell surface disappeared and the lamellar plica was also visible.

The assessment of DC morphology under the microscope showed that the majority of cells were adherent and arranged in clusters, the morphology of the cells was normal, and the cells were irregular in shape, including stars, circles, and spindles (Figure 1(b)). The scanning electron microscope examination revealed that the cell bodies had an irregular shape and rough surface, the microvilli on the cell surface disappeared, and the lamellar plica was also visible (Figure 1(c)). In addition, the DC surface markers CD11c and CD14 were detected by FACS, and the proportion of CD11c^+^ CD14^+^ DCs reached 97.2% after GM-CSF and IL-4 treatment (Figure 1(a)).

### 3.2. TCDD Activated DCs through AhR

**Figure 2:**
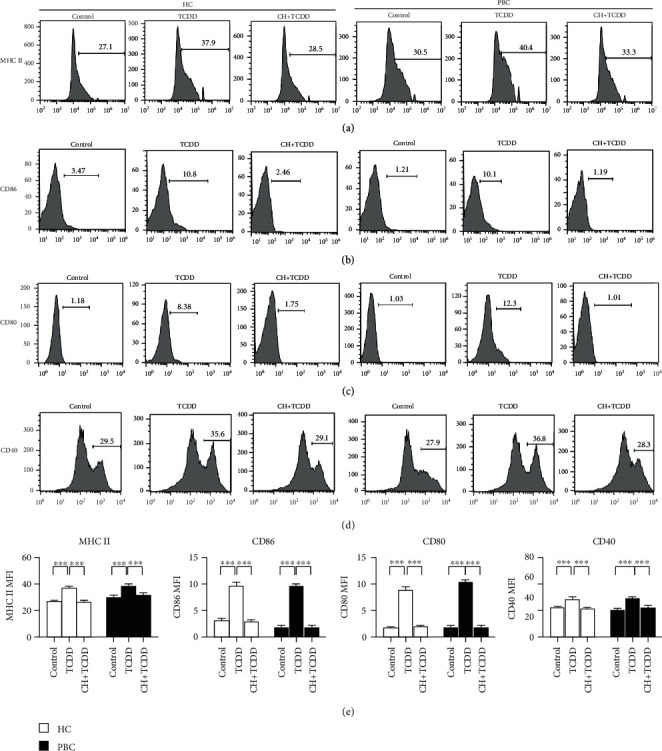
DCs are activated through AhR agonists TCDD. DCs from HCs and PBC patients were treated with 10 nM TCDD (TCDD group) and 10 *μ*M CH223191+10 nM TCDD (CH+TCDD group) for 48 h and stained for MHC II, CD86, CD80, and CD40 mAbs analyzed by FACS. The differences of MFI of MHC II (a), CD86 (b), CD80 (c), and CD40 (d) were analyzed in different groups by statistical analysis (e). In both the healthy control and PBC patients, compared to the blank control and CH+TCDD group, the MFI of MHC II, CD86, CD80, and CD40 were increased. Results are presented as mean ± SEM. ^∗^Significantly higher than the control, *p* < 0.05 (^∗^*p* < 0.05, ^∗∗^*p* < 0.01, and ^∗∗∗^*p* < 0.001).

DCs were divided into three groups: untreated DCs as a blank control, 10 nM TCCD-treated DCs as an experimental group, and 10 *μ*M CH223191+10 nM TCDD-treated DCs as a negative control group. The changes in MHC II, CD86, CD80, and CD40 were assessed through changes in MFI after 2 days of treatment. As shown in Figure 2, the MFI of MHC II (a), CD86 (b), CD80 (c), and CD40 (d) increased in the TCDD group compared with the control and TH+TCDD groups in both patients with PBC and HCs (*p* < 0.05). In addition, CH223191 as an antagonist of dioxin-induced AhR activation suggested that DCs were activated by AhR [16].

### 3.3. TCDD Stimulated DCs to Secrete Cytokines by AhR

**Figure 3:**
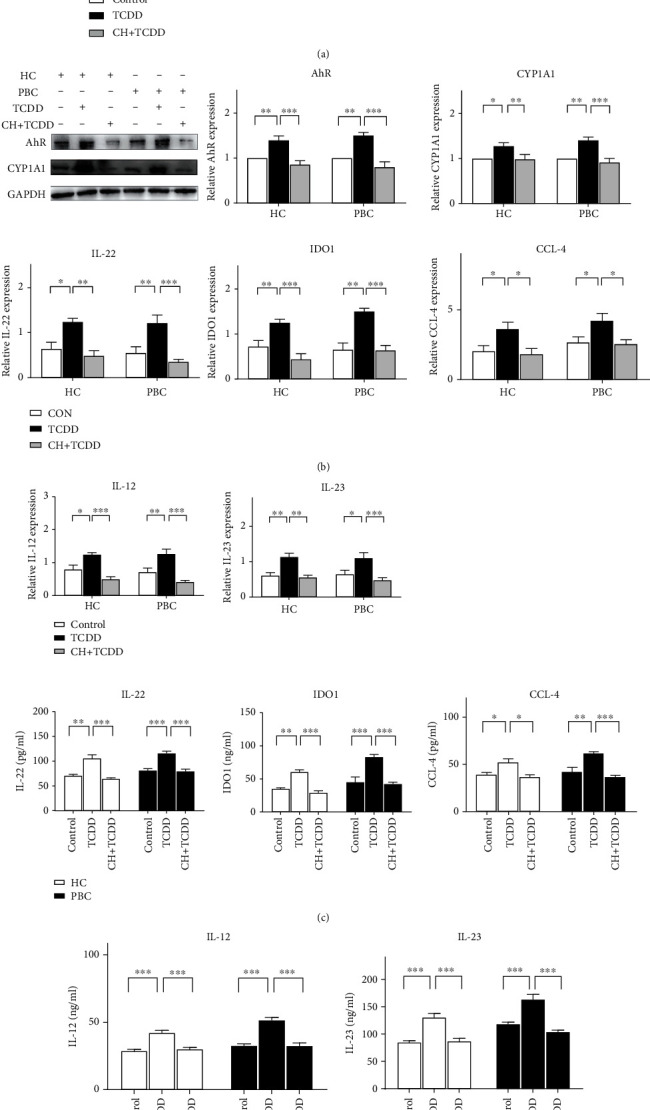
DCs are activated through AhR ligands. DCs were stimulated by with 10 nM TCDD (TCDD group) and 10 *μ*M CH223191+10 nM TCDD (CH+TCDD group) for 48 h. DCs of HC and PBC patients were obtained and analyzed the expression of AhR and CYP1A1 by qRT-PCR (a) and Western Blots (b). The expression of AhR and CYP1A1 were significantly increased after TCDD treatment. After TCDD stimulated DCs, the expressions of IL-22, IDO1, CCL-4, IL-12, and IL-23 of the TCDD group were higher than other groups by qRT-PCR (c) and ELISA (d). Results are presented as mean ± SEM. ^∗^Significantly higher than the control, *p* < 0.05 (^∗^*p* < 0.05, ^∗∗^*p* < 0.01, and ^∗∗∗^*p* < 0.001).

As shown in Figure 3, the mRNA expression level of AhR and CYP1A1 was higher in the TCDD group compared with the control and CH+TCDD groups in both HCs and patients with PBC (Figure 3(a)). Meanwhile, TCDD-treated DCs had a significant increase in the protein levels of AhR and CYP1A1 (Figure 3(b)). The RNA and protein expression of related cytokines and chemokines were detected by qRT-PCR (Figure 3(c)) and ELISA (Figure 3(d)), respectively, to examine the effect of TCDD on DCs activated by AhR. After stimulation of DCs by TCDD, the mRNA expression levels of IL-22, indoleamine 2,3-dioxygenase (IDO1), CCL-4, IL-12, and IL-23 were higher in the TCDD group than in the control and CH+TCDD groups. The ELISA results showed that the expression levels of IL-22, IDO1, CCL-4, IL-12, and IL-23 were higher in the TCDD group than in other groups.

### 3.4. TCDD-Treated DCs Altered the Expression of IFN-*γ*, IL-17, T-bet, and ROR*γ*t in Naïve CD4^+^ T Cells

**Figure 4:**
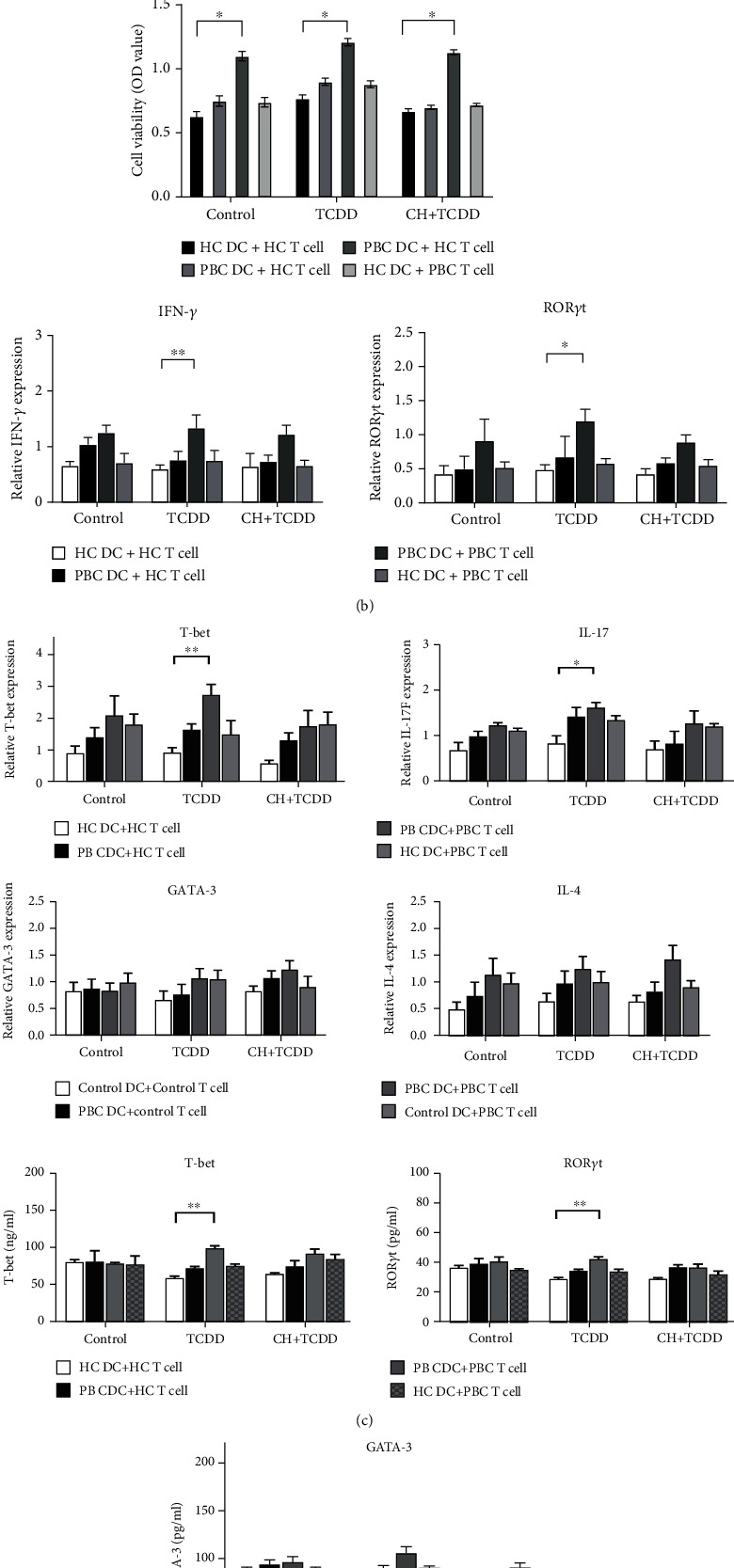
TCDD-treated DCs altered the expression of IFN-*γ*, IL-17, T-bet, and ROR*γ*t in naïve CD4^+^ T cells. DCs were cocultured with PBC patients or HC naïve CD4^+^ T cells for 3 days. (a) Naïve CD4^+^ T cells surface markers were detected by FACS and the proportion of CD4^+^ CD45RA^+^ naïve CD4^+^ T cells reached 97.8%. (b) CCK-8 detected the naïve CD4^+^ T cell proliferation differences between different groups. (c)After coculture, the expressions of IFN-*γ*, IL-17, T-bet, and ROR*γ*t of the PBC DC+PBC T cell group were higher than those of the HC DC+HC T cell group in RNA level. (d) Then, AhR-activated DCs altered the expression of T-bet and ROR*γ*t in naïve CD4^+^ T cells at protein levels using ELSA kit. Data are expressed as mean ± SEM. ^∗^Significantly higher than control, *p* < 0.05 (^∗^*p* < 0.05, ^∗∗^*p* < 0.01, and ^∗∗∗^*p* < 0.001).

Naïve CD4^+^ T cells surface markers CD4^+^CD45RA^+^ were detected by FACS, and the proportion of CD4^+^CD45RA^+^ naïve CD4^+^ T cells reached 97.8% (Figure 4(a)). CCK-8 was used to detect the proliferation of naïve CD4^+^ T cells after coculture. Whether in the control, TCDD, or CH + TCDD group, the proliferation of CD4^+^ T cells in the PBC DC+PBC T cell group was higher than that in the HC DC+HC T cell group. However, the proliferation of naïve CD4^+^ T cells in the PBC DC+HC T cell and HC DC+PBC T cell groups was not significantly different from that in the HC DC+HC T cell group (Figure 4(b)). The mRNA and protein levels of related cytokines were detected by qRT-PCR (Figure 4(c)) and ELISA (Figure 4(d)), respectively, to examine the effect of TCDD-treated DCs on naïve CD4^+^ T cells. After TCDD-treated DCs were cocultured with naïve CD4^+^ T cells, the mRNA expression levels of IFN-*γ* (1.33 ± 0.24), T-bet (2.72 ± 0.33), IL-17 (1.92 ± 0.12), and ROR*γ*t (1.19 ± 0.20) were higher in the PBC DC+PBC T cell group than in the HC DC+HC T cell group (0.59 ± 0.08, 0.89 ± 0.24, 0.84 ± 0.18, and 0.68 ± 0.07; *p* < 0.01, *p* < 0.01, *p* < 0.05, and *p* < 0.05, respectively). The protein expression levels of T-bet (99.08 ± 3.9) and ROR*γ*t (42.29 ± 1.75) were also higher in the PBC DC+PBC T cell group than in the HC DC+HC T cell group (59.04 ± 2.96 vs. 28.96 ± 1.23, *p* < 0.01). However, no significant differences were found in the levels of T cell differentiation-related cytokines in other groups.

### 3.5. TCDD-Treated DCs Affected Naïve CD4^+^ T Cell Differentiation

**Figure 5:**
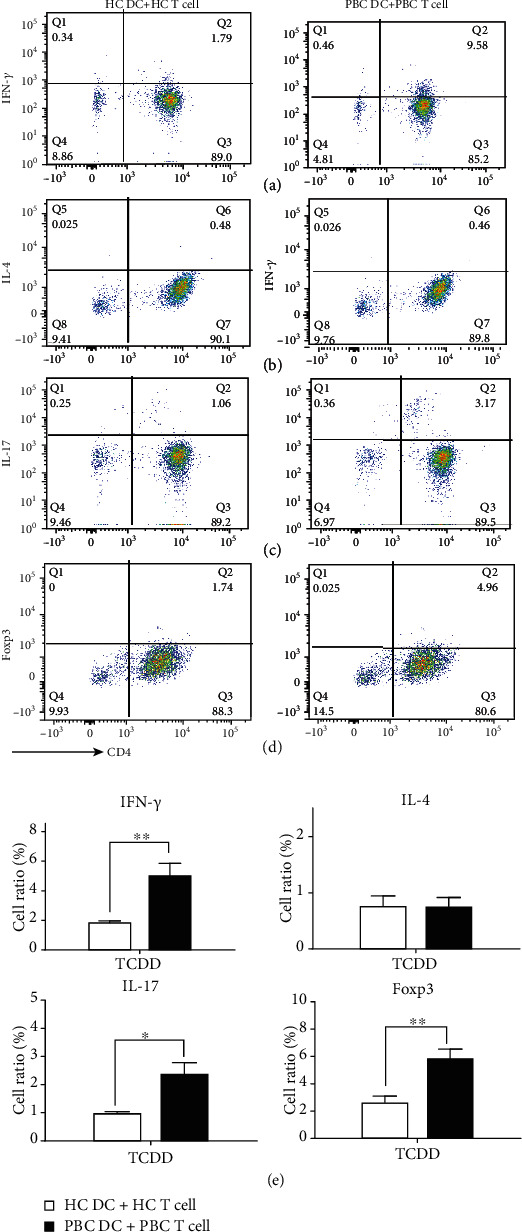
TCDD-treated DCs affect naïve T cell differentiation. TCDD-treated DCs were cocultured with naïve CD4^+^ T cells, and the cell ratios of Th1, Th2, and Th17 were detected by FACS. In the PBC group, the differences of cell ratio (%) of IFN-*γ* (a), IL-4 (b), IL-17 (c), and Foxp3 (d) were analysis in the different groups by statistical analysis (e). The cells ratio of Th1 and Th17 of the PBC DC+PBC T cell group was higher than the HC DC+HC T cell group. The cells ratio of Th2 showed no significant differences between the HC and PBC groups. Data are expressed as mean ± SEM (^∗^*p* < 0.05, ^∗∗^*p* < 0.01, and ^∗∗∗^*p* < 0.001).

The mRNA expression levels of naïve CD4^+^ T cell differentiation-related cytokines in the CH+TCDD group did not differ. Hence, the levels of IFN-*γ*, IL-4, IL-17, and Foxp3 in the TCDD group were detected using FACS. As shown in Figure 5, in the PBC group, after TCDD-treated DCs were cocultured with naïve CD4^+^ T cells, the cell ratio of Th1 was lower in the HC DC+HC T cell group than in the PBC DC+PBC T cell group (*p* < 0.01). The cell ratio of Th17 was higher in the PBC DC+PBC T cell group than in the HC DC+HC T cell group (*p* < 0.05). The cell ratio of Th2 showed no significant differences between the HC and PBC groups. Meanwhile, in the HC group, there have no meaningful change (Fig. S1). The result indicated that TCDD-treated DCs affected the differentiation of Th1 and Th17.

## 4. Discussion

PBC is an autoimmune disease caused by a genetic susceptibility to the external environment [17]. AhR is a transcription factor activated by a number of environmental factors and regulates the activity of immune cells [18]. However, a loss of functional T cells in PBC results in the loss of immune regulation on effector CD4^+^ T cells, thereby disrupting immune tolerance and promoting the occurrence of PBC [19]. The effect of AhR activation on potent toxins such as TCDD is known for its ability to promote Th1 and Th17 [20]. In this study, the expression levels of MHC II, CD86, CD80, and CD40 increased through TCDD stimulation. DCs trigger T cells through MHC II, CD86, CD80, and CD40 presentation and costimulation, subsequently directly activating T cells [21]. CD40 activation induced Fas-dependent apoptosis and NF-kappaB/AP-1 signaling in human intrahepatic biliary epithelial cells [22]. The present study demonstrated that AhR agonist promoted DC activation and induced a regulatory phenotype.

In this study, the expression levels of AhR and CYP1A1 increased after TCDD stimulation, indicating that TCDD activated AhR in DCs [23]. In animal experiments, environmental pollutants promoted Th17 differentiation by activating AhR to secrete CYP1A1 [24]. The function of DCs and their immunoregulatory role in T cell differentiation depend on the regulation and expression of cytokines and chemokines [25]. Previous studies found that environmental pollutants activated AhR in DCs and secreted IL-22 to promote T cell differentiation into Th17 cells [26]. IDO1 has immunoregulatory effects associated with tryptophan metabolism on T cell function [27]. CCL-4 can indirectly favor the development of T cells through suppressing both T-bet expression and T cell differentiation [28]. Meanwhile, DCs produce IL-23, which induces the production of IL-17 by Th17 [29]. IL-23 is vital in promoting the proliferation and effector function of Th17 cells, which are characterized by the expression of IL-17 family cytokines. Studies suggested that IL-12 promoted the differentiation of Th1 cells and induced IFN-*γ* production [30]. In this study, the expression levels of IL-22, CCL-4, IDO1, IL-23, and IL-12 were higher in DCs of patients with PBC after TCDD stimulation. However, the effects of these cytokines on naïve CD4^+^ T cells are complex, and the specific mechanism underlying their exact role in PBC needs further exploration.

Different CD4^+^ T lymphocyte subgroups have different roles in PBC [31]. In this study, the expression of IFN-*γ*, T-bet, IL-17, and ROR*γ*t increased in the PBC DC+PBC T cell group was compared with the HC DC+HC T cell group. The changes in these indicators were related to the dysfunction of CD4^+^ T cell differentiation [17]. IFN-*γ* and IL-17 were crucial in the initiation and regulation of disease and accounted largely for the autoimmune pathology. Th1 and Th17 cells are primarily localized around the damaged interlobular bile ducts in PBC [32]. The present study found that TCDD-treated DCs could secrete IL-12 and IL-23 to promote Th1 and Th17 differentiation. It was hypothesized that TCDD, as an alien biomass, could stimulate the differentiation of Th1 and Th17 through DCs, leading to the aggregation of Th1 and Th17 in bile duct epithelial cells and promoting the occurrence of PBC. How Th1 and Th17 affect bile duct epithelial cells needs further exploration.

In addition, a total of four coculture systems were used in this study. The results showed that naïve CD4^+^ T cells of patients with PBC were more likely to be activated by TCDD-treated DCs of patients with PBC, leading to abnormal differentiation. This finding was consistent with the environmental susceptibility of patients with PBC. Hence, environmental problems are an important part of the pathogenesis of PBC.

It was reasonable to speculate the existence of multiple pathways through which DCs proliferated and differentiated into T cells when presenting T cell antigens. In addition, the expression of cytokines and chemokines in the control group was not significantly changed after coculture. It was speculated that patients with PBC had more pronounced responses to xenobiotics, making individuals more susceptible to autoimmune disease. Therefore, the specific mechanism through which cytokines and chemokines mediate these effects warrants further investigation.

To sum up, the imbalance between Th1 cells and Th17 cells might be the cause for the disorder of liver autoimmune function in patients with PBC and the induction of AMAs. The evaluation of the relationship between Th1 and Th17 cells might provide a new therapeutic approach to the pathogenesis of autoimmune liver disease, so as to prevent and treat the disease by intervening with the functioning of Th1 and Th17 cells.

## 5. Conclusions

This study successfully explored the effects of TCDD-activated DCs on naïve CD4^+^ T cell differentiation in patients with PBC. TCDD-activated DCs could secrete cytokines to promote Th1 and Th17 differentiation in patients with PBC. Thus, AhR was vital in the pathogenesis of PBC. AhR on DCs might be a potential therapeutic target for treating PBC.

## Figures and Tables

**Table 1:** Primer sequences.

Primer	Primer sequences
GAPDH	F: GGAGCGAGATCCCTCCAAAAT R: GGCTGTTGTCATACTTCTCATGG
h-IFN-*γ*	F: GGAGACCATCAAGGAAGACATGA R: AGTTCAGCCATCACTTGGATGAG
h-IL4	F: TGTGCTCCGGCAGTTCTACA R: CCTTCACAGGACAGGAATTCAAG
h-CCL4	F: AGCGCTCTCAGCACCAATG R: GCTTCTTTTGGTTTGGAATACCA
h-AhR	F: CGTGGGTCAGATGCAGTACAA R: TGATGAAGTGGCTGAAGATGTGT
h-IL-12	F: GATGGCCCTGTGCCTTAGTAGT R: TGCAGGTCATCACCTTCAATATG
h-IL-23	F: CAACAGTCAGTTCTGCTTGCAAA R: AGTTGGCTGAGGCCCAGTAG
h-IDO1	F:CTGAGCACCTTCTTTCCCTTCA R: TGCCTTTCCAGCCAGACAA
h-T-bet	F: GCCTACCAGAATGCCGAGATT R: ATCTCCCCCAAGGAATTGACA
h-IL-22	F: TCTGATGAAGCAGGTGCTGAAC R: TGCAGGTCATCACCTTCAATATG
h-ROR-*γ*t	F: GCCTACCAGAATGCCGAGATT R: GAGGACCTGGGAGTAGATGAGGTA
h-GATA-3	F: CACAGAAGGCAGGGAGTGTGT R: AGCCTTCGCTTGGGCTTAAT
h-CYP1A1	F: CACAGAAGGCAGGGAGTGTGT R: AGCCTTCGCTTGGGCTTAAT
h-IL-17	F: CGCGTTTCCATGTCACGTAA R: GATGTCTTCCTTTCCTTGAGCATT

## Data Availability

All data generated or analyzed during this study are included in this article.

## Supplementary Materials

Supplementary materials Figure S1: TCDD-treated DCs affect naïve T cell differentiation. TCDD-treated DCs were cocultured with naïve CD4+ T cells and the cell ratios of Th1, Th2, and Th17 were detected by FACS. In the HC group, the differences of cell ratio (%) of IFN-*γ* (A), IL-4 (B), IL-17 (C), and Foxp3 (D) were analyzed in different groups by statistical analysis (E). There have no meaningful change. Data are expressed as mean ± SEM^∗^*p* < 0.05, ^∗∗^*p* < 0.01, and ^∗∗∗^*p* < 0.001). Table S1: biochemical data for PBC patients and healthy controls.
