# Supplementary material for: Effects of 2,3,7,8-Tetrachlorodibenzo-p-dioxin on T Cell Differentiation in Primary Biliary Cholangitis
Source: Biomed Res Int. 2020 Aug 24;2020:1754975. doi: 10.1155/2020/1754975 (PMC7468604; doi:10.1155/2020/1754975)
Supplement: Supplementary materials — Figure S1: TCDD-treated DCs affect naïve T cell differentiation. TCDD-treated DCs were cocultured with naïve CD4+ T cells and the cell ratios of Th1, Th2, and Th17 were detected by FACS. In the HC group, the differences of cell ratio (%) of IFN-γ (A), IL-4 (B), IL-17 (C), and Foxp3 (D) were analyzed in different groups by statistical analysis (E). There have no meaningful change. Data are expressed as mean ± SEM∗p < 0.05, ∗∗p < 0.01, and ∗∗∗p < 0.001). Table S1: biochemical data for PBC patients and healthy controls. [file 1754975.f1.docx]

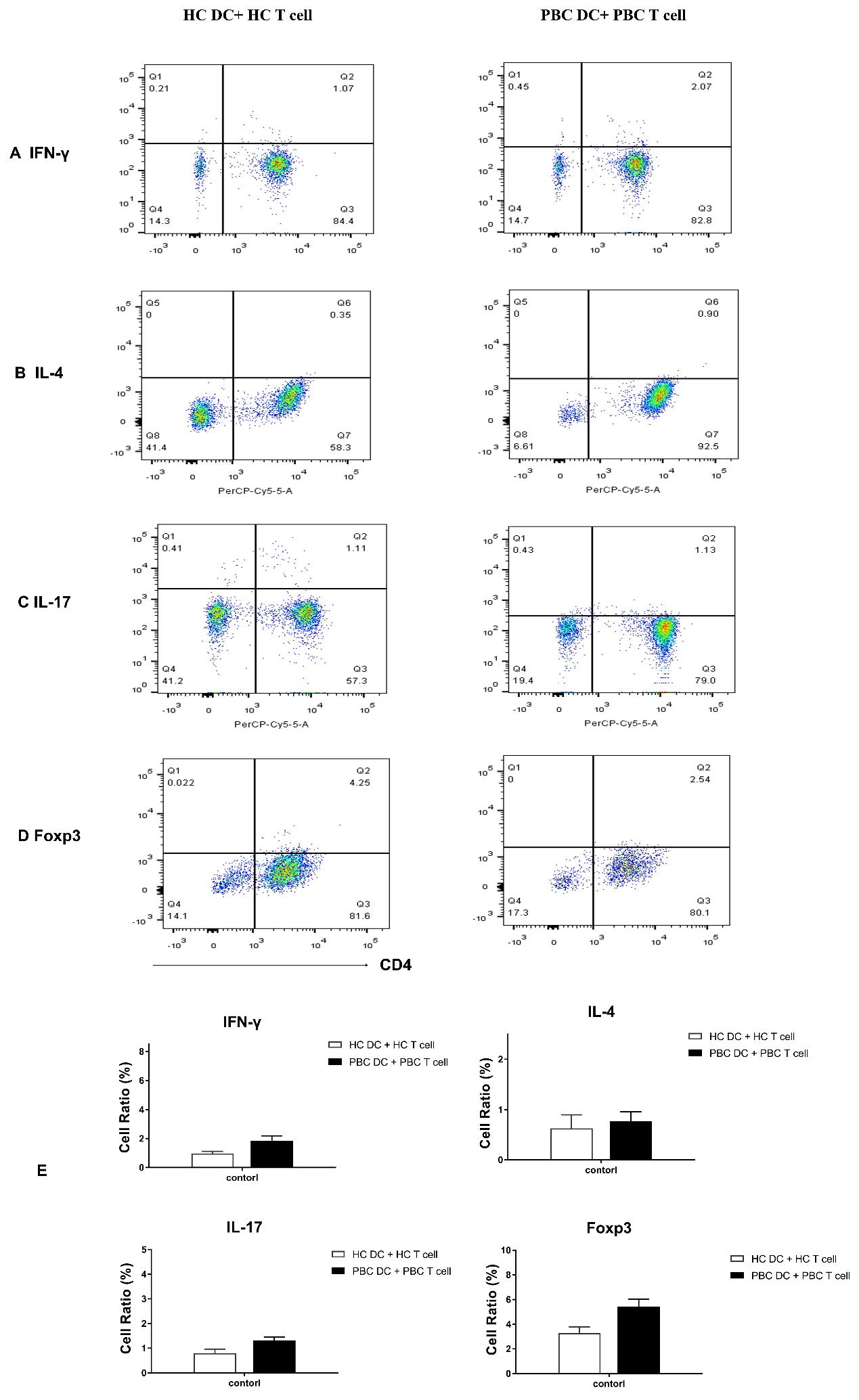


FigS1. TCDD-treated DCs affects naïve T cell differentiation. TCDD-treated DCs were co-culture with naïve CD4+ T cells and the cell ratios of Th1, Th2 and Th17 were detected by FACS. In HC group, the differences of cell ratio (%) of IFN-γ (A), IL-4 (B), IL-17 (C) and Foxp3 (D) were analysis in difference group by statistic (E). There have no meaningful change Data are expressed as mean ± SEM. * p < 0.05; ** p < 0.01; ***p<0.001).

Table. S1 Biochemical data for PBC patients and healthy controls

| Indexes PBC HC |
| --- |
| Female（%） 100% 100%  Old 59.90±6.24 55.18±5.06  AMA-M2（%） 100% 0%  ALT(IU/L) 117.17±58.82 24.31±9.92  AST(g/L) 74.72±19.06 31.56±5.32  TBIL(IU/L) 1.68±0.04 1.09±0.02  ALP(IU/L) 217.68±32.04 112.78±6.09  IgM(g/L) 5.18±1.31 2.39±0.52 |

Abbreviations: AMA-M2, Anti-mitochondrial antibody-M2; ALT, Alanine aminotransferase; AST, Aspartate aminotransferase; TBIL, total bilirubin; ALP, alkaline phosphatase; IgM, Immunoglobulin M.
